# Alleviative effects of plant growth-promoting rhizobacteria on salt-stressed rice seedlings: mechanisms mediated by rhizosphere microbiota and root exudates

**DOI:** 10.3389/fpls.2025.1661074

**Published:** 2025-10-08

**Authors:** Jinjun Wang, Xinru Gao, Mingliang Yang, Yang Zhang, Yinglong Chen

**Affiliations:** ^1^ College of Environmental Science and Engineering, Yangzhou University, Yangzhou, China; ^2^ Key Laboratory of Arable Land Quality Monitoring and Evaluation, Ministry of Agriculture and Rural Affairs, Yangzhou University, Yangzhou, China; ^3^ Key Laboratory of Saline-Alkali Soil Reclamation and Utilization in Coastal Areas, The Ministry of Agriculture and Rural Affairs of China, Yangzhou University, Yangzhou, China; ^4^ Jiangsu Key Laboratory of Crop Genetic and Physiology, Yangzhou University, Yangzhou, China; ^5^ Jiangsu Key Laboratory of Crop Cultivation and Physiology, Yangzhou University, Yangzhou, China; ^6^ Jiangsu Co-Innovation Center for Modern Production Technology of Grain Crops, Yangzhou University, Yangzhou, China; ^7^ Research Institute of Rice Industrial Engineering Technology, Yangzhou University, Yangzhou, China

**Keywords:** PGPR, salt stress, rice seedlings, rhizobacterial community, root exudates

## Abstract

**Introduction:**

Salt stress represents a critical abiotic constraint that severely impedes plant growth and agricultural productivity. While plant growth-promoting rhizobacteria (PGPR) demonstrate potential in enhancing plant salt tolerance, their precise mechanisms remain incompletely elucidated. This study systematically investigates the mechanistic basis by which PGPR inoculation ameliorates salt stress in rice seedlings through modulation of rhizosphere microbiota and root exudate profiles.

**Methods:**

We inoculated rice seedlings with five monocultures (*Bacillus* sp., *Providencia* sp., etc.) and a synthetic consortium (T6) under salt stress conditions. Growth parameters, rhizobacterial communities (via 16S rRNA sequencing), and root exudates (untargeted metabolomics) were comparatively analyzed against uninoculated controls (CK).

**Results:**

PGPR inoculation significantly promoted rice seedling growth under salt stress. Treatments T2-T6 exhibited substantial increases in key biomass parameters—including root length, plant height, and dry weight—relative to the CK control. Concurrently, elevated chlorophyll content and enhanced photosynthetic efficiency were observed. Inoculated plants also displayed significantly higher activities of antioxidant enzymes (Superoxide dismutase, Peroxidase, Catalase activity) (SOD, POD, CAT) and proline (Pro) accumulation in both leaves and roots, coupled with a marked reduction in Malondialdehyde, indicating effective mitigation of oxidative damage. PGPR inoculation altered rhizosphere bacterial community composition, reducing overall alpha-diversity. Notably, the relative abundance of dominant bacterial phyla (e.g., *Proteobacteria*, *Acidobacteriota*) and beneficial genera (e.g., *Subgroup_7*, *Lysobacter*) increased significantly. These microbial shifts showed positive correlations with improved plant physiological status, suggesting a synergistic role in promoting seedling growth under salt stress. Root exudate metabolomics revealed a substantial number of differentially abundant metabolites in inoculated plants compared to CK, encompassing lipids, hormones, and signaling molecules. Crucially, the production of these specific exudates correlated with the enrichment of dominant bacterial taxa in the rice rhizosphere. Metabolic pathway analysis indicated significant enrichment primarily within Nucleotide metabolism and Purine metabolism pathways (belonging to the Metabolism superclass) and ABC transporter pathways (within Environmental Information Processing). The T6 consortium treatment induced enrichment across a significantly greater number of key metabolic pathways compared to single-strain inoculations.

**Discussion:**

PGPR inoculation enhances rice seedling growth and salt tolerance by: (1) optimizing rhizosphere microbiota (enriching dominant phyla and beneficial genera); (2) recruiting stress-mitigating microbial consortia; and (3) stimulating root exudates enriched in nucleotide/purine metabolism and ABC transporters. The superior efficacy of the T6 consortium underscores the advantage of synergistic microbial interactions. Collectively, these findings reveal plant-microbe-metabolite mechanisms underlying PGPR-mediated salt tolerance, providing a foundation for developing salinized soil remediation strategies.

## 1 Introduction

Statistics from the Food and Agriculture Organization (FAO) of the United Nations show that the global area of salinized land has reached 1.381 billion hectares (representing approximately 10.7% of the global land area) (Yu, 2025). As an important existing and potential arable resource, saline-alkali soil has significant potential for development and utilization. Traditional saline-alkali land management mainly relies on physical methods (such as irrigation for salt leaching) and chemical improvement, yet these are constrained by methodological limitations, high costs, and high recurrence risk. Contemporary research has shifted toward bio-agronomic synergistic strategies, establishing sustainable ecological reclamation systems through revegetation (e.g., halophyte cultivation) integrated with microbial remediation (e.g., PGPR inoculation) (Yang et al., 2016; Luo et al., 2021; Wang et al., 2024; Wang, 2024).

PGPR are widely distributed and diverse, encompassing groups such as bacteria, fungi, and actinomycetes, with *Pseudomonas*, *Bacillus*, and *Rhizobium* as dominant groups (Lugtenberg and Kamilova, 2009; Luo et al., 2021). Due to their dual functions of promoting plant growth and controlling pests/diseases, as well as advantages such as ecological environmental protection and strong environmental compatibility, PGPR have been widely applied and attracted significant attention in soil remediation (Arora and Kumar, 2019; Luo et al., 2021). As core components of plant-microbe interaction systems, PGPR participate in stress resistance regulation of host plants through multiple mechanisms such as metabolic interaction and signal transduction. For example, nutrient deficiency in saline-affected soils constitutes a major constraint limiting plant growth and yield enhancement. Rhizosphere microorganisms play a pivotal role in enhancing plant nutrient acquisition through multifaceted mechanisms, *Bacillus aquimaris* can increase nitrogen content in wheat leaves under salt stress (Upadhyay and Singh, 2015); *Azospirillum brasilense* and *Pseudomonas fluorescens* synergistically promote soil nitrogen mineralization and significantly enhance rice biomass. Inoculation with salt-tolerant PGPR elevates phytohormone levels in plant roots, consequently mitigating stress-imposed impairments under saline conditions. *B. cereus* regulates the plant cytokinin (CTK) signal transduction system under drought stress, coordinating CTK transport in root-shoot communication (Liu et al., 2015); *Bacillus licheniformis* increases the relative biomass of chrysanthemums by 35-42% and reduces root Na+ accumulation by 28-33% under 200 mM NaCl stress by synthesizing ABA (Cheng et al., 2017). Additionally, PGPR can enhance plant salt-alkali resistance by synthesizing osmolytes. Studies have shown that *Bacillus subtilis* improved proline synthesis capacity through overexpression of *proB* and *proA* genes, simultaneously enhancing its own salt tolerance and plant osmotic tolerance (Wu et al., 2018). These studies have revealed the multiple action mechanisms of PGPR in plant stress resistance regulation and their important application value in agricultural ecology.

Recent breakthroughs in functional research on Plant Growth-Promoting Rhizobacteria (PGPR) have revealed their significant biocontrol potential in crop cultivation and soil remediation. PGPR orchestrate host plant responses to abiotic stresses through multifaceted metabolic pathways, demonstrating versatile roles in plant-microbe interactions. Substantial evidence confirms that diverse PGPR taxa—including Bacillus, Chryseobacterium, Pseudomonas, Azospirillum, Achromobacter, Aeromonas, and Acetobacter (Etesami and Maheshwari, 2018)—enhance plant salinity tolerance via synergistic mechanisms. Under saline soil conditions, PGPR inoculation improves stress resilience metrics, promotes biomass accumulation, optimizes nutrient cycling efficiency, and stabilizes agricultural ecosystems. Metabolomic profiling by Ali et al (Ali et al., 2022). demonstrated that Enterobacter cloacae PM23 upregulates the proline synthesis gene ProDH, triggering osmolyte accumulation (e.g., proline, glycine betaine) in maize seedlings. This process maintains cellular turgor under moderate salt stress (100 mM NaCl). Complementary transcriptomic analysis (Girma et al., 2022) revealed that Klebsiella sp. KBG6.2 mitigates sodium chloride-induced oxidative damage (150 mM) in rice by activating indole-3-acetic acid (IAA) signaling while suppressing reactive oxygen species (ROS) burst in root apices.

This study integrates metagenomic sequencing and plant untargeted metabolomics technologies to systematically clarify the mechanism by which salt-tolerant PGPR inoculation promotes rice growth in salinized soils by regulating rhizospheric microbial community structure and root exudates. On the one hand, it enhances our theoretical understanding of PGPR enhancing crop salt tolerance; on the other hand, it provides experimental evidence for developing salinized soil improvement technologies based on salt-tolerant rice-microbe synergistic systems.

## 2 Materials and methods

### 2.1 Pot experiments and preparation of inoculants

The pot experiments with rice were conducted in a rooftop experimental facility at Yangzhou University (Yangzhou, China). Air-dried and sieved soil was filled into plastic pots (15 cm height × 15 cm diameter) at 2 kg per pot. Seven treatments were set up: CK (non-inoculated control), T1 (*Bacillus* sp.), T2 (*Providencia* sp.), T3 (*Planococcus* sp.), T4 (*Pseudoclavibacter* sp.), T5 (*Dietzia* sp.), and T6 (a mixture of the above five strains), with three replicates per treatment (21 pots in total). Rice seedlings with uniform growth were selected and transplanted into the pots (two hills per pot, four plants per hill). Seven days later, bacterial suspensions were evenly irrigated around the rice roots at an inoculation concentration of 10^8^ CFU/g soil, while CK was watered with an equal volume of sterile water. A water layer depth of 2–3 cm was maintained by daily observation and replenishment with deionized water (Li et al., 2025). Physiological and biochemical indices of rice were measured after four weeks.

Typical single colonies were selected and inoculated into 5 mL of LB liquid medium, then cultured in a 28°C shaking incubator at 180 rpm for 12 h to prepare primary seed solutions. Subsequently, the primary culture was transferred to 100 mL of LB medium at a volume ratio of 1:100 and continuously cultured at 30°C with the same rotation speed for 12 h. After cultivation, bacterial pellets were collected by centrifugation (4°C, 8000×g, 10 min), and the supernatant was discarded. The pellets were resuspended in sterile water to an appropriate concentration.

### 2.2 Determination of physiological and biochemical indices

After four weeks of growth, intact rice plants were harvested, and root soils were rinsed with tap water. Residual water was blotted dry with filter paper, and the plant height and main root length were measured and recorded. Roots and shoots were separated, blanched in a constant-temperature drying oven at 115°C for 30 min, and then dried at 80°C to constant weight. Dry weights of roots and shoots were weighed using an electronic balance.

Chlorophyll content in rice leaves was determined by ethanol extraction colorimetry (Li, 2000). For antioxidant enzyme activity assays (Wang and Huang, 2015), SOD activity was measured by the nitroblue tetrazolium method, CAT activity by the guaiacol method, and POD activity by the UV absorption method in rice tissues (roots and leaves). MDA content was determined by the thiobarbituric acid colorimetry, and free proline content was measured by sulfosalicylic acid extraction–acid ninhydrin colorimetry (Wang and Huang, 2015) in roots and leaves.

### 2.3 Collection and analysis of root exudates

Root exudates of each treatment were collected in the fourth week of the experiment. Roots were first rinsed with sterile water, transferred to a 1 L beaker wrapped with tin foil, and immersed in sterile water. After continuous culture for 24 h, the solution was filtered through a sterile 0.45 μm microporous membrane. The collected solution was lyophilized by a freeze dryer to obtain dry powder of root exudates, which was stored in a sterile airtight container at -20°C. Subsequent LC-MS untargeted metabolomics analysis was commissioned to Majorbio Bio-Pharm (Shanghai, China), and the obtained data were further processed.

Metabolites that passed quality control were functionally annotated using the KEGG database, categorizing them based on their involvement in biological pathways or molecular functions. Orthogonal partial least squares-discriminant analysis (OPLS-DA) was conducted to statistically compare metabolic profiles among six inoculation treatments (T1–T6) and the control group (CK), systematically elucidating the regulatory effects of plant growth-promoting rhizobacteria (PGPR) on the rice root exudome. Significantly differential metabolites (top 30 ranked by variable importance in projection (VIP) scores with VIP ≥ 1) were selected for hierarchical clustering analysis using Euclidean distance and complete linkage algorithms. A composite visualization integrating a heatmap with VIP bar plots was generated to display metabolite distribution patterns, expression profiles, and statistical parameters (VIP values and *P*-values) across experimental groups.

Comparative metabolomic profiling between inoculated and non-inoculated groups identified differentially abundant metabolites exhibiting significant up- and down-regulation. Core metabolites were structurally classified using the Human Metabolome Database (HMDB), and KEGG pathway enrichment analysis was performed to identify significant pathways (false discovery rate [FDR] < 0.05) containing ≥2 mapped metabolites. Finally, Spearman’s rank correlation analysis was employed to elucidate association networks between dominant microbial taxa and root-secreted metabolites.

### 2.4 Processing and sequencing analysis of rhizospheric soil samples

During plant harvest, rhizospheric soil samples were collected using standardized methods. After intact root systems were removed, large soil clumps were shaken off, and soils tightly attached to root surfaces were collected and immediately stored in a -80°C freezer.

After DNA extraction, PCR amplification was performed for the V1-V9 variable regions of the bacterial 16S rRNA gene using the forward primer 27F (5’-[Barcode]AGAGTTTGATCMTGGCTCAG-3’) and reverse primer 1492R (5’-ACCTTGTTACGACTT-3’). The PCR reaction system contained 15 μL of 2× Phanta Max Master Mix (Vazyme), 0.5 μM of each primer, 10–30 ng of template DNA, and ddH_2_O up to 30 μL. The thermal cycling parameters were: initial denaturation at 95°C for 5 min; 30 cycles of 94°C for 30s (denaturation), 56°C for 30 s (annealing), and 72°C for 45s (extension); final extension at 72°C for 10 min; and storage at 4°C. The amplified products were verified by 2% agarose gel electrophoresis (120V, 40 min), and specific bands (450–550 bp) were excised, purified using the AxyPrep DNA Gel Extraction Kit (Axygen), and eluted with 30 μL of sterile EB buffer (10 mM Tris-HCl, pH 8.5). Microbiome data analysis was performed based on the QIIME2 platform (version 2022.11). In the sequence processing stage, demultiplexing assigned paired-end reads to sample-specific barcodes, and primer sequences were excised using Cutadapt (v3.7). The DADA2 pipeline performed quality filtering (Q-score ≥ 20), denoising with removal of non-overlapping sequences, paired-end merging requiring a minimum 12-bp overlap, and chimera detection against the SILVA 138 reference database. High-quality sequences were clustered at 100% similarity to generate amplicon sequence variants (ASVs), and a feature table (ASV-sample abundance matrix) was constructed. For taxonomic annotation and filtering, ASVs were classified via BLASTn alignment (E-value ≤ 1e^−5^) against the Greengenes 13_8 database (99% identity threshold). Low-abundance ASVs (relative abundance < 0.001%, equivalent to < 10 reads per million) were removed to generate a filtered feature table. In community profiling, taxonomic composition was visualized using stacked bar plots (phylum/class levels) generated with ggplot2 (v3.4.2) in R. Rarefaction curves confirmed adequate sequencing depth (Good’s coverage > 99%). Features were subsampled to 8,500 sequences per sample (5th percentile of minimum sequencing depth) to standardize library sizes.

For diversity analysis: Alpha diversity indices (Chao1 for richness; Shannon for evenness) were computed in QIIME2; intergroup differences were visualized via boxplots;Beta diversity analysis used weighted UniFrac distances for principal coordinate analysis (PCoA). Intergroup dissimilarities were assessed by Bray-Curtis-based PERMANOVA (999 permutations; P < 0.05). For differential analysis and integration: LEfSe identified differentially abundant taxa across groups. Spearman’s rank correlation revealed associations between dominant microbiota and rice physiological indices. Metabolic pathways with significant alterations (adj. P < 0.05, |log_2_FC| > 1) were characterized with key contributing taxa.

## 3 Results

### 3.1 Effects of PGPR inoculation on rice growth under salt stress

**Figure 1:**
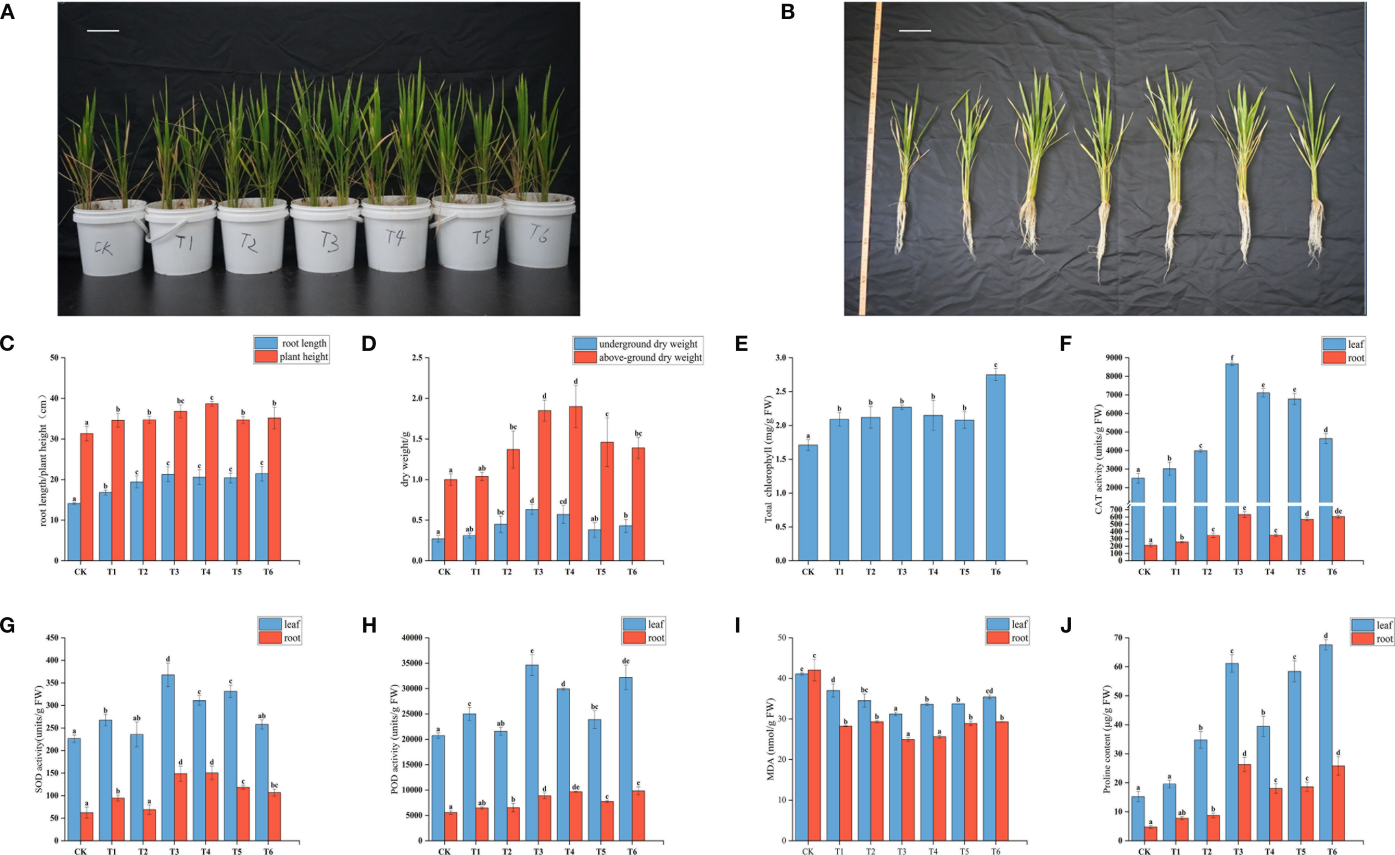
Effects of different treatment groups on **(A, B)** rice seedling growth, **(C)** root length and plant height,**(D)** above-ground and below-ground dry weight, **(E)** total chlorophyll content, **(F-H)** SOD, CAT, and POD content in rice leaves and roots, **(I, J)** MDA and proline content in rice leaves and roots.

As shown in **Figures 1A, B**, compared with CK, all inoculated treatments promoted rice growth. Root length significantly increased (p<0.05) in all T1-T6 treatments compared to CK, with the largest increase (51.77%) observed in T6 (**Figures 1B, C**). Plant height significantly increased (p<0.05) in T1-T4 and T6 treatments, but not in T5 (10.76% increase, not significant). The T4 treatment showed the greatest increase in plant height (23.43%) (**Figure 1B**). In the T1 treatment, there were no significant changes in the dry weight of roots and shoots. However, the dry weight of shoots significantly increased in T2-T6 treatments, with the T3 treatment showing the greatest increase (133.33%) (**Figure 1D**). The dry weight of roots in the T4 treatment was 1.9 times that of CK (**Figure 1D**).

Chlorophyll, a key catalyst for plant photosynthesis, is an important indicator of photosynthetic efficiency and growth status. The chlorophyll content in all T1-T6 treatments was significantly higher than in CK (*p* < 0.05). The T6 treatment showed the most significant effect, with an increase of 60.82% compared to CK (**Figure 1E**), indicating that mixed inoculation with PGPR has a clear advantage in improving total chlorophyll content in rice leaves under salt stress.

To assess the effects of different treatments on rice defense capacity, the activities of antioxidant enzymes (POD, SOD, CAT), MDA content, and Pro content were measured in rice leaves and roots. The antioxidant enzyme activities in both leaves and roots of all treatment groups were higher than in CK (**Figures 1F–J**), indicating that PGPR inoculation enhances the antioxidant capacity of rice seedlings under salt stress. Overall, most treatments showed significant increases compared to CK. The largest increase in SOD activity in leaves was observed in T3 (62.40%), while in roots, T4 showed the greatest increase (141.10%). For CAT activity, T3 had the greatest increase in both leaves (245.66%) and roots (199.15%). For POD activity, T3 showed the greatest increase in leaves (67.16%), and T4 in roots (73.26%).

MDA content, a key indicator of oxidative stress, increases when plants are subjected to stress due to intensified lipid peroxidation. The MDA content in both leaves and roots of T1-T6 treatments was significantly reduced (p<0.05) compared to CK (**Figure 1I**), indicating that PGPR inoculation effectively alleviates salt stress in rice. The inhibitory effects on MDA content varied among treatments, with T3 showing the most prominent effect: MDA content in leaves and roots decreased by 24.04% and 40.65%, respectively. The reduction in MDA content in roots was significantly greater than in leaves for all T1-T6 treatments (p<0.05), indicating that PGPR inoculation provides more significant protective effects on roots.

Free Pro is an important osmolyte in plant responses to abiotic stress, and its accumulation is closely associated with stress tolerance. The Pro content in both leaves and roots of T1-T6 treatments increased compared to CK, with significant increases in T2-T6 (p<0.05) (**Figure 1J**), indicating that PGPR inoculation enhances rice salt tolerance by promoting Pro accumulation. The T3 and T6 treatments showed the most significant increases: leaf Pro content increased by 302.10% and 344.31%, respectively, and root Pro content by 456.03% and 445.24%, respectively. The T2, T4, and T5 treatments also exhibited significant increases in Pro content in both leaves and roots (p<0.05).

### 3.2 Effects of PGPR inoculation on the rhizosphere bacterial community of rice under salt stress

Multi-omics analysis of the rhizosphere soil microbial community of rice using 16S rRNA amplicon sequencing showed that after rigorous quality control procedures, the total number of raw sequencing data reached 522,660, with the number of raw sequencing data per sample ranging from 73,120 to 75,989. After chimera filtering and sequence optimization, the total number of high-quality sequences was 492,998, with individual sample valid sequence counts ranging from 68,807 to 71,759. Sequencing read lengths, after quality assessment, ranged from 681 to 1,471 base pairs, meeting the requirements for subsequent analysis. At the same time, we uploaded the sequencing sequences to the NCBI database with the accession number PRJNA1304747.

**Figure 2:**
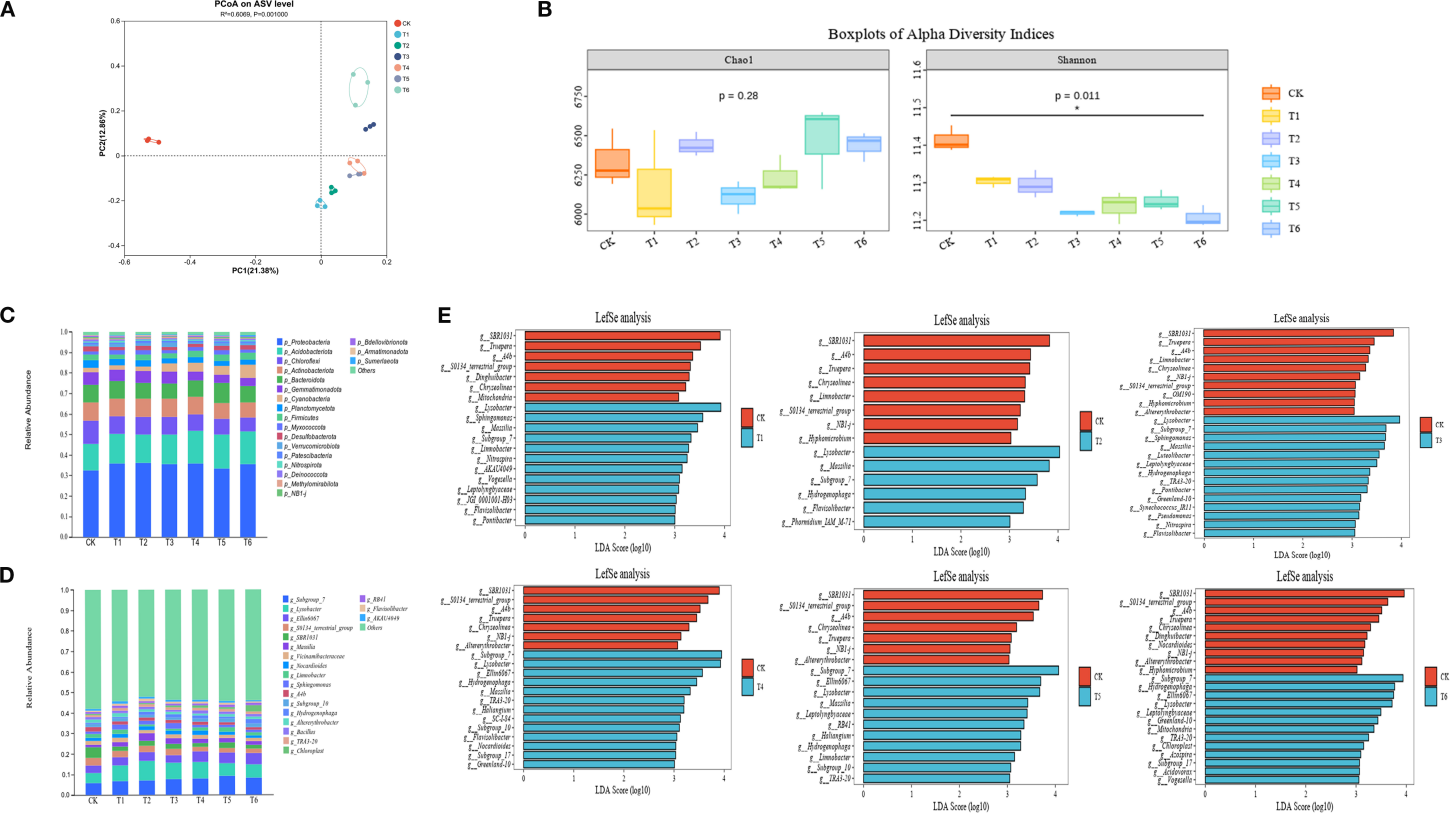
Rice rhizosphere bacterial community **(A)** PcoA plot and **(B)** Alpha diversity box plot, [**(C)** phylum level; **(D)** genus level] rice rhizosphere bacterial community composition and **(E)** genus level LefSe analysis.

Principal Coordinates Analysis (PCoA) based on Bray-Curtis dissimilarity (**Figure 2A**) revealed clear separation between inoculated (T1-T6) and CK groups. PC1 (21.38%) and PC2 (12.86%) collectively explained 34.24% of community variance, confirming significant PGPR-induced restructuring of the rhizosphere microbiome under salt stress.

Alpha diversity analysis (**Figure 2B**) showed significantly reduced Shannon indices in all inoculated treatments (T1-T6) versus CK (*p* < 0.05), indicating decreased species diversity and evenness. The Chao1 index showed no significant differences among treatments (*p* > 0.05), demonstrating that PGPR inoculation primarily affects community evenness rather than species richness.

Community composition analysis identified dominant bacterial phyla across all treatments (**Figure 2C**): *Proteobacteria*, *Acidobacteriota*, *Chloroflexi*, *Actinobacteriota*, *Bacteroidota*, and *Gemmatimonadota*. At genus level (**Figure 2D**), core taxa included: *Subgroup_7* (6.07–9.23%), *Lysobacter* (4.63–9.52%), *Ellin6067* (3.49–5.70%), *S0134_terrestrial_group* (2.06–3.65%), and *SBR1031* (1.79–4.98%). PGPR inoculation consistently enriched beneficial genera (*Subgroup_7*, *Lysobacter*, *Ellin6067*, *Massilia*, *Sphingomonas*) while suppressing *S0134_terrestrial_group*, *SBR1031*, *Limnobacter*, and *A4b* relative to CK.

Linear discriminant analysis ffect Size (LEfSe) (LDA > 3; **Figure 2E**) identified treatment-specific biomarkers, with shared enrichment of beneficial taxa (*Subgroup_7*, *Lysobacter*, *Massilia*, *Hydrogenophaga*, *Ellin6067*, *Sphingomonas*) across multiple inoculated treatments.

Finally, OTU abundance profiles derived from high-throughput sequencing were mapped to the KEGG database to characterize functional attributes of rhizobacterial communities across treatment groups. At KEGG Pathway Level 3, PGPR inoculation elevated the abundances of key bacterial metabolic pathways in saline-stressed rhizosphere soils, including:Ansamitocin biosynthesis (ko01051); Vancomycin antibiotic biosynthesis (ko01055); C5-Branched dibasic acid metabolism (ko00660); Valine, leucine and isoleucine biosynthesis (ko00290); Fatty acid biosynthesis (ko00061); Pantothenate and CoA biosynthesis (ko00770); Synthesis and degradation of ketone bodies (ko00072); Lipoic acid metabolism (ko00785).

(**Table 1**).

**Table 1:** Functional annotation of KEGG pathway 3 for rhizosphere soil bacterial communities of rice in different treatment groups.

Pathway level 3	CK	T1	T2	T3	T4	T5	T6
ko01051 Biosynthesis of ansamycins	2.66	2.78	2.79	2.76	2.77	2.82	2.78
ko01055 Biosynthesis of vancomycin group antibiotics	2.15	2.22	2.23	2.23	2.25	2.29	2.23
ko00290 Valine, leucine and isoleucine biosynthesis	2.12	2.18	2.18	2.18	2.17	2.18	2.17
ko00660 C5-Branched dibasic acid metabolism	1.87	1.93	1.92	1.91	1.92	1.93	1.91
ko00061 Fatty acid biosynthesis	1.83	1.90	1.89	1.88	1.90	1.9	1.88
ko00770 Pantothenate and CoA biosynthesis	1.65	1.68	1.68	1.67	1.66	1.67	1.66
ko00072 Synthesis and degradation of ketone bodies	1.61	1.66	1.66	1.66	1.65	1.60	1.61
ko00785 Lipoic acid metabolism	1.56	1.61	1.60	1.61	1.60	1.61	1.61
ko00471 D-Glutamine and D-glutamate metabolism	1.63	1.61	1.61	1.60	1.58	1.61	1.59
ko00550 Peptidoglycan biosynthesis	1.51	1.51	1.51	1.51	1.51	1.52	1.51
ko00521 Streptomycin biosynthesis	1.53	1.50	1.51	1.50	1.51	1.52	1.50
ko00473 D-Alanine metabolism	1.49	1.46	1.46	1.45	1.46	1.47	1.45
ko00970 Aminoacyl-tRNA biosynthesis	1.46	1.44	1.44	1.43	1.43	1.45	1.43
ko02030 Bacterial chemotaxis	1.36	1.38	1.41	1.36	1.37	1.38	1.39
ko04112 Cell cycle – Caulobacter	1.36	1.35	1.34	1.34	1.33	1.34	1.33
ko00670 One carbon pool by folate	1.35	1.34	1.34	1.33	1.33	1.34	1.33

### 3.3 Differences in the composition of rice root exudates

**Figure 3:**
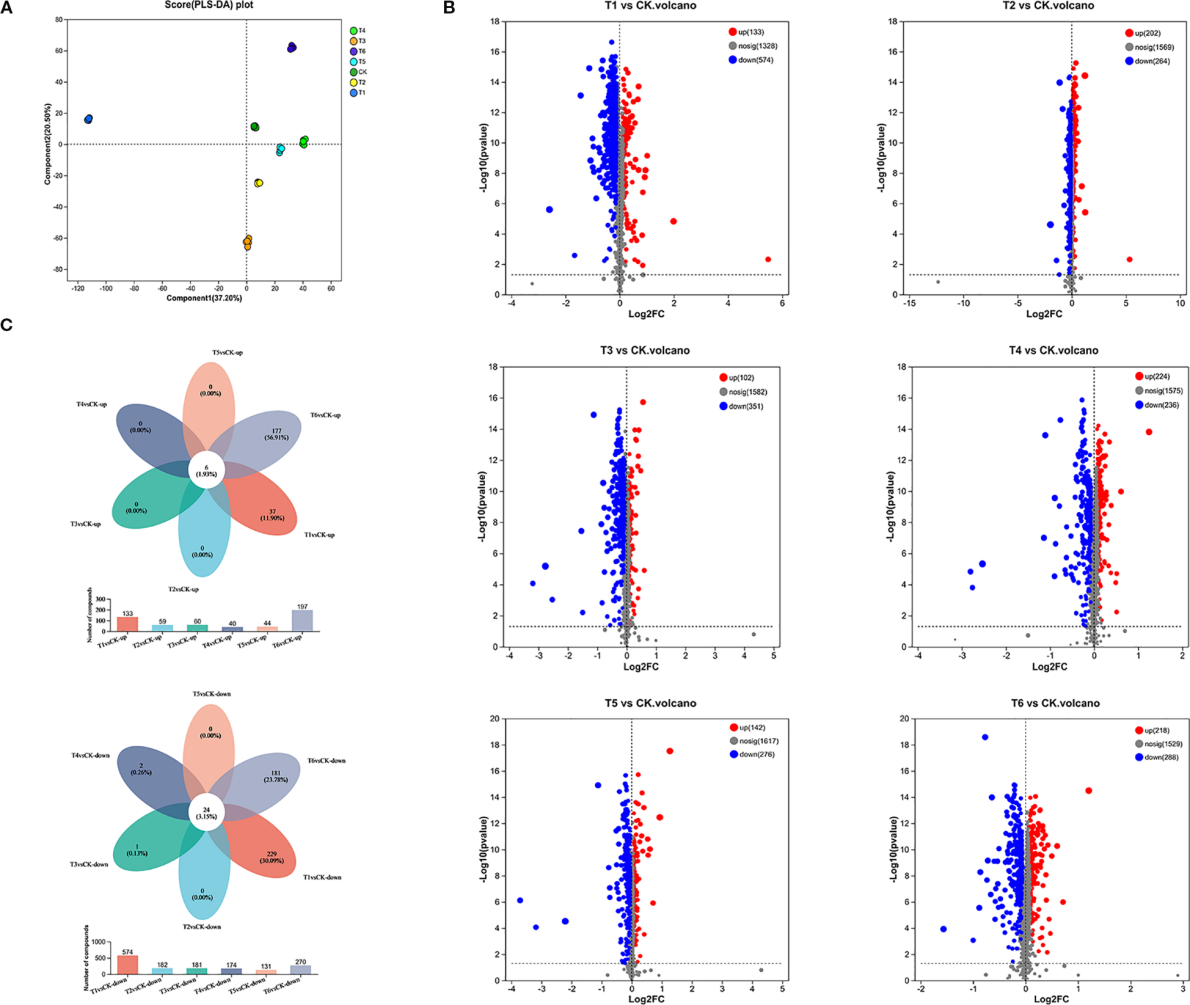
**(A)** PLS-DA of root exudates, **(B)** Total ion model differential metabolite volcano plot for each differential grouping, **(C)** Venn diagram of differentially expressed exudates in rice under different treatment groups.

To investigate the effects of PGPR inoculation on rice root exudates under salt stress and their association with rhizosphere microbiome functional regulation, we analyzed exudates from six treatments. We identified 2,029 metabolites, comprising 1,379 cationic and 650 anionic metabolites. Partial Least Squares Discriminant Analysis (PLS-DA) revealed significant intergroup differences in exudate composition (**Figure 3A**).

Volcano plots of differentially expressed metabolites (DEMs) indicated a predominant downregulation trend in all treatments relative to CK (**Figure 3B**). T1 exhibited the highest number of downregulated metabolites (n=574). These results confirm that PGPR inoculation significantly alters root exudate composition, with downregulation being the dominant response.

Through Venn diagram analysis of common and unique differential exudates among different comparison treatment groups, the results were shown as follows: Six common upregulated DEMs, with 37 and 177 unique to T1 and T6 (vs. CK), respectively;Twenty-four common downregulated DEMs, with 229 and 181 unique to T1 and T6 (vs. CK), respectively. (**Figure 3C**). Functional annotation classified these DEMs into lipids, hormones and transmitters, steroids, vitamins and cofactors, and nucleic acids (**Table 2**). Downregulated DEMs in T1-T6 (vs. CK) were predominantly lipids, hormones and transmitters, and steroids, while upregulated DEMs were primarily nucleic acids and peptides.

**Table 2:** Annotated table of classification of plant compounds of differential secretion.

Categorization	T1		T2		T3		T4		T5		T6	
	Up	Down	Up	Down	Up	Down	Up	Down	Up	Down	Up	Down
Lipids	4	14	2	3	4	2	0	1		2	2	3
Organic acids	1	0	0	0	0	0	0	0	0	0	1	0
Nucleic acids	1	5	1	0	1	0	0	0	1	0	1	6
Carbohydrates	0	2	0	0	0	0	0	0	0	0	0	3
Hormones and transmitters	2	7	1	1	1	1	1	3	1	1	1	1
Steroids	2	6	1	3	1	2	1	3	1	2	1	2
Vitamins and cofactor	0	3	0	1	1	0	0	0	1	0	1	2
Peptides	1	0	1	0	1	0	0	0	0	0	4	0

**Figure 4:**
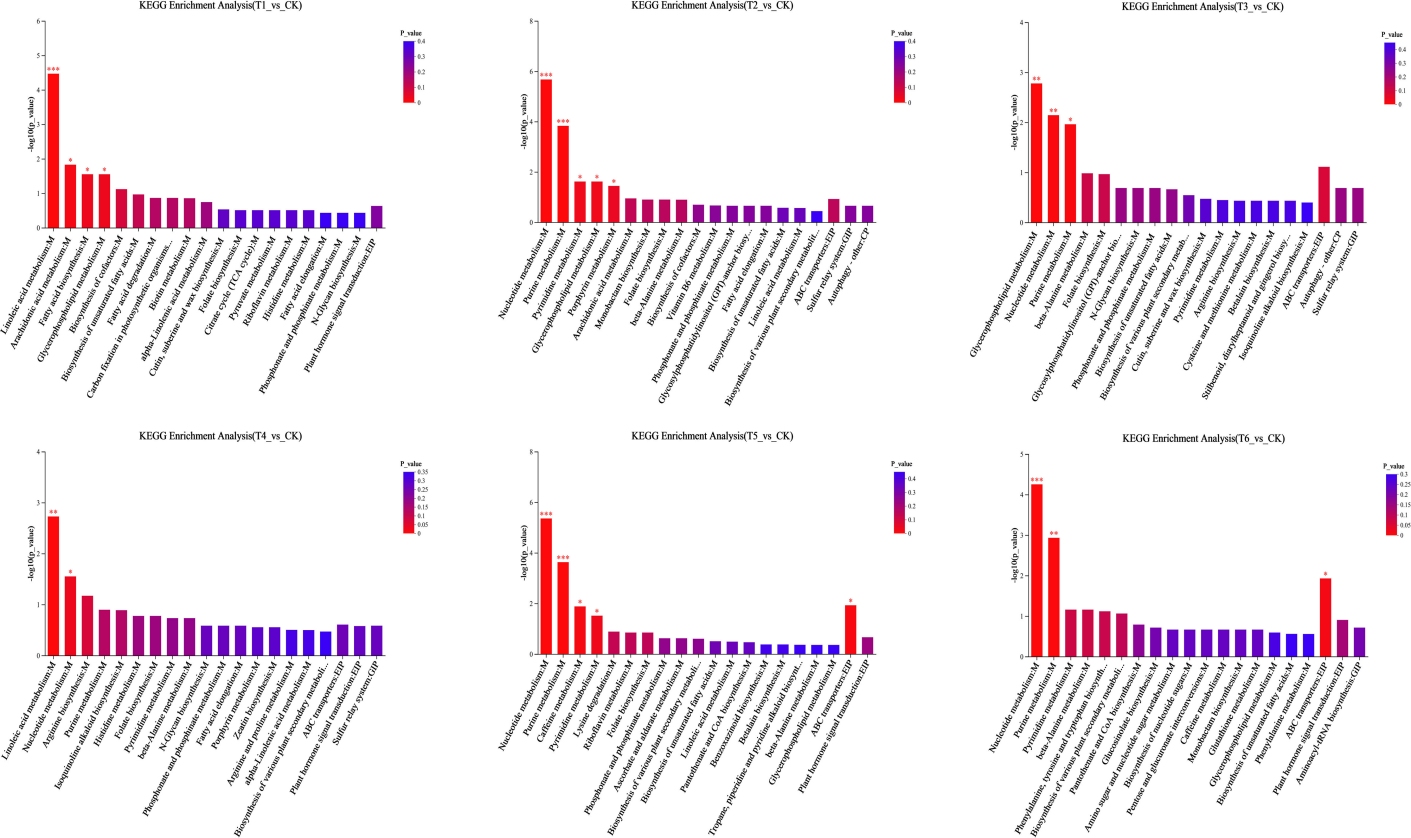
KEGG enrichment analysis.

Pathway enrichment analysis indicated that key DEMs in T1-T6 were primarily enriched in nucleotide metabolism, purine metabolism (Metabolism), and ABC transporters (Environmental Information Processing) (**Figure 4**). Specifically:T1 exhibited significant enrichment (*p* < 0.001) in linoleic acid metabolism (Metabolism).T2 and T3 were enriched in glycerophospholipid metabolism (Metabolism).The mixed inoculation (T6) showed significantly more enriched pathways than single strains, including tryptophan metabolism, phenylalanine/tyrosine/tryptophan biosynthesis (Metabolism), and plant hormone signal transduction (Environmental Information Processing).

### 3.4 Correlation analysis between rice rhizosphere soil bacterial communities and physiological indicators, root exudates

**Figure 5:**
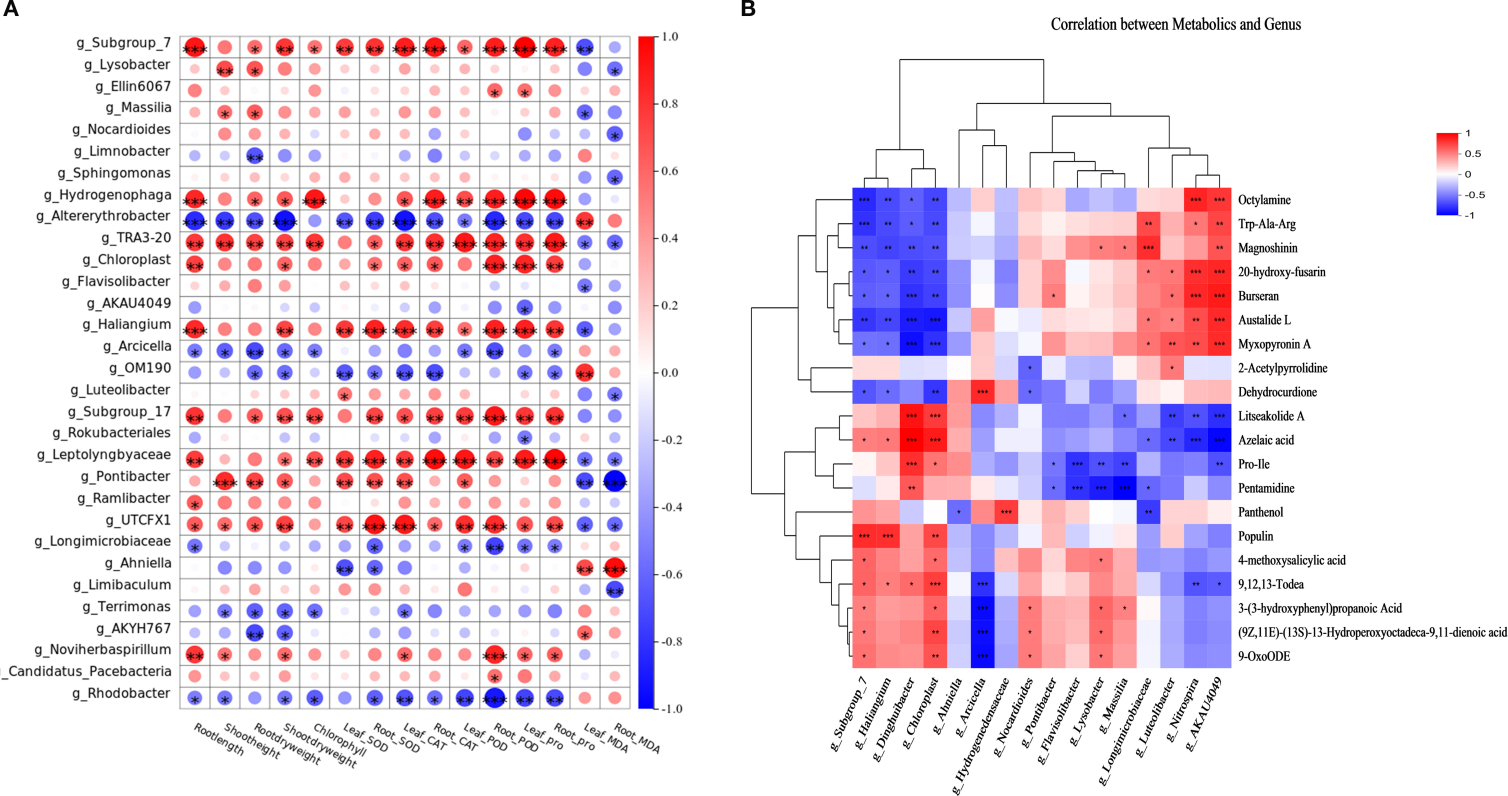
**(A)** Heat map of correlation between genus-level enriched taxa of rice rhizosphere bacteria and rice physiological indicators;**(B)** Heatmap of correlation analysis between rice root secretion and rhizosphere bacterial genus level enrichment taxa.

Correlation analysis revealed significant associations between key soil bacterial groups and rice physiological indicators and root exudates. We concluded that:

Beneficial taxa (*Subgroup_7*, *Subgroup_17*, *Hydrogenophaga*, *Haliangium*, *Leptolyngbyaceae*) showed significant positive correlations (*p* < 0.05) with growth parameters (root length, plant height, shoot/root biomass, chlorophyll) and stress markers (SOD, CAT, POD, Proline), while exhibiting negative correlations with MDA (**Figure 3A**).Conversely, *SBR1031*, *A4b*, *Altererythrobacter*, *Arcicella*, and *Rhodobacter* displayed inverse correlation patterns (**Figure 5A**).

*Subgroup_7*, *Subgroup_17*, *Hydrogenophaga*, *TRA3-20*, *Haliangium*, *Leptolyngbyaceae*, etc., showed significant positive correlations with growth parameters (root length, plant height, above- and below-ground dry weight, chlorophyll content) and stress markers (SOD, CAT, POD, Pro), and significant. negative correlations with MDA. In contrast, *SBR1031*, *A4b*, *Altererythrobacter*, *Arcicella*, *Rhodobacter*, exhibited inverse correlations (**Figure 5A**).

Metabolite-specific relationships revealed three distinct microbial functional groups:

Protective Taxa: *Subgroup_7* and *Haliangium* exhibited significant negative correlations with stress-associated metabolites (octylamine, tryptophyl-alanyl-arginine, magnoshinin, 20-hydroxyfusarin, burseran, austalide L, myxopyronin A, dehydrocurdione) while showing positive correlations with beneficial compounds (litseakolide A, azelaic acid, populin, 9,12,13-trihydroxy-10E-octadecenoic acid). Stress-Associated Taxa: *Longimicrobiaceae*, *AKAU4049*, *Luteolibacter*, and *Nitrospir*a demonstrated inverse patterns - positively correlating with the aforementioned stress metabolites but negatively correlating with litseakolide A, azelaic acid, prolyl-isoleucine, and pentamidine. Dual-Role Taxa: *Lysobacter*, *Massilia*, and *Flavisolibacter* displayed selective associations: positive correlations with magnoshinin and 3-(3-hydroxyphenyl)propanoic acid, yet negative correlations with prolyl-isoleucine and pentamidine (**Figure 5B**).

## 4 Discussion

Salt stress is an important environmental factor that severely limits global crop productivity. The use of appropriate beneficial PGPR represents a greener, cleaner approach to improving crop production and environmental resilience under salt stress. This study systematically investigated the effects of PGPR inoculation on rice seedling growth, rhizosphere microbial communities, and root exudate metabolism under salt stress. The results align with existing research and provide multidimensional evidence for elucidating the salt tolerance regulatory mechanisms of PGPR.

### 4.1 Effects of different treatments on rice growth

We found that PGPR alleviated salt stress damage by enhancing the antioxidant system and osmotic regulation function, consistent with the theory proposed by Fiodor et al (Fiodor et al., 2021). that “PGPR inoculation may increase nutrient absorption and accumulation by promoting root development.” In the experiment, different PGPR inoculation treatments significantly increased SOD, CAT, and POD activities in rice leaves and roots, consistent with the results reported by Jha et al (Jha and Subramanian, 2013) and Chen et al (Chen et al., 2024). Meanwhile, MDA content was significantly reduced, alleviating oxidative stress caused by salt stress, in line with the findings of Han et al (Han et al., 2014). and Chauhan et al (Chauhan et al., 2019), indicating that PGPR activated the enzymatic defense network to remove excess ROS. Notably, under mild salt stress (e.g., soil salinity of 0.2–2.2‰), PGPR significantly enhanced chlorophyll content more effectively than under severe stress, possibly because the low-salt environment did not exceed the metabolic regulatory threshold of PGPR. Overall, PGPR inoculation improved chlorophyll content in rice leaves under salt stress (Xin et al., 2011; Gao, 2014). In this experiment, proline content in rice leaves and roots increased significantly across all treatment groups, consistent with the findings of Jha et al (Jha and Subramanian, 2013). As a core component of osmotic regulation, proline accumulation was enhanced by PGPR through the activation of the OsP5CS1/2 gene, aligning with the molecular mechanisms observed in transgenic rice studies (Wang et al., 2021).

### 4.2 Differences in rhizosphere bacteria under salt stress

Our study also observed that PGPR inoculation led to a decrease in rhizosphere bacterial alpha diversity (Shannon index), consistent with the community clustering results from PCoA. Specifically, under salt stress, PGPR preferentially promoted the colonization of beneficial bacteria, leading to enhanced community structure selectivity and a reduction in overall α-diversity (Tahir et al., 2015; Jing et al., 2018). The survival of exogenous bacteria depends on environmental compatibility; when soil conditions such as pH and temperature are suboptimal, even inoculants with high viable cell counts may exhibit poor survival rates and consequently fail to outcompete indigenous microbial populations. In most cases, inoculation enhances plant nutrient acquisition and promotes growth—both directly and indirectly—by altering rhizosphere soil pH and modulating microbiome composition and interactions (da Cunha et al., 2024).

At the phylum level, *Proteobacteria*, *Acidobacteriota*, *Chloroflexi*, and *Actinobacteriota* were the four dominant phyla across all treatments, similar to the findings of Sarathambal et al (Sarathambal et al., 2022), confirming that PGPR inoculation does not recruit dominant bacterial groups in a host plant-specific manner. The enrichment of these dominant bacterial groups carries clear functional implications. *α- and γ-Proteobacteria*—exemplified by nitrogen-fixing genera such as Rhizobium (α-class) and Pseudomonas (γ-class)—convert atmospheric nitrogen into plant-available forms, significantly enhancing plant growth through biological nitrogen fixation. In saline-alkaline soils, these *Proteobacteria* facilitate rapid rhizospheric organic matter degradation, exhibiting competitive dominance in carbon-rich environments. Their abundance correlates positively with soil organic matter content, and their metabolic plasticity contributes to plant stress tolerance under adverse conditions (Rilling et al., 2018; Das et al., 2020; Anzalone et al., 2022). *Acidobacteriota* abundance frequently exhibits negative correlations with soil nitrogen/phosphorus availability. This phylum likely employs oligotrophic strategies to outcompete copiotrophs under nutrient-depleted conditions, contributing to carbon cycling through decomposition of recalcitrant organic compounds (e.g., cellulose) and thereby enhancing soil fertility sustainability (Zhang et al., 2023). *Chloroflexi* contributes to phytoremediation of contaminated soils under heavy metal-polluted or hypersaline conditions through sulfur cycling and anaerobic metabolic pathways. In contrast, *Actinobacteriota* plays a role in enhancing soil nutrient content, inhibiting the growth of pathogenic microorganisms, and promoting plant growth (Bhattacharyya et al., 2018; Guarino et al., 2020). *Proteobacteria* and *Actinobacteriota* dominate nitrogen and phosphorus transformation, while *Chloroflexi* and *Acidobacteriota* participate in carbon cycling, collectively maintaining root zone nutrient balance and resisting environmental fluctuations. This functional division confirms that PGPR exhibits cross-host conservation in recruiting dominant microbial communities.

At the genus level, genera such as *Subgroup_7*, *Lysobacter*, *Massilia*, and *Hydrogenophaga* were significantly enriched in the rice rhizosphere. This directed regulation formed a specific microbial community architecture that was more conducive to the adaptive growth of rice seedlings under salt stress. Among these, *Subgroup_7*, as the core group of *Acidobacteriota*, prefers acidic environments, participates in the decomposition of recalcitrant organic matter (such as cellulose), regulates soil carbon cycling, and stabilizes soil aggregates by secreting extracellular polysaccharides, thereby improving the rhizosphere microenvironment (Yang et al., 2021). *Lysobacter*, *Massilia*, and *Hydrogenophaga* (all belonging to *Proteobacteria*) construct the defense and nutritional network of the rhizosphere microbiome through functions such as decomposing recalcitrant carbon sources, inhibiting pathogens, promoting nutrient cycling, and enhancing plant resistance (Ruth et al., 2015). Additionally, the relative abundance of these groups showed a significant positive correlation with rice physiological and biochemical indicators, promoting seedling growth under salt stress.

Functional gene analysis further revealed enrichment of the pantothenate and CoA biosynthesis pathway (ko00770) at KEGG Level 3, indicating PGPR may enhance microbial oxidative stress adaptation by modulating redox cofactor metabolism (Ilangumaran and Smith, 2017). The upregulation of ansamitocin (ko01051) and vancomycin-type antibiotic biosynthesis (ko01055) genes suggests PGPR inoculation induces antagonistic interactions. These antibiotics not only suppress phytopathogens but also reinforce the competitive dominance of beneficial taxa (e.g., Bacillus, Pseudomonas) through rhizosphere microbiome restructuring (Khanghahi et al., 2025). Notably, although salinity stress typically reduces Actinobacteriota abundance, PGPR-secreted antibiotics may preserve functional guild equilibrium—a mechanism corroborated in rapeseed rhizospheres by Świątczak et al (Swiatczak et al., 2023).

### 4.3 Differences in the composition of rice root exudates under salt stress

Analysis using the OPLS-DA model indicated that PGPR treatment significantly altered the composition of root exudates, with the number of DEMs ranging from 418 to 707 across treatment groups—primarily downregulated, suggesting that PGPR reshapes the rhizosphere microenvironment by inhibiting specific metabolic pathways. The chemical classification of differentially secreted compounds showed that lipids and steroids were predominantly downregulated, while peptides, vitamins, and cofactors were predominantly upregulated. Nucleic acids were predominantly downregulated in T1 and T6 but upregulated in T2–T5. Peptides (Trp-Ala-Arg) play critical roles in plant root secretions as signal regulators, growth promoters, stress resistance enhancers, and influencers of rhizosphere microorganisms. For example, brassinosteroids collaborate with other plant hormones (such as auxins) to regulate growth and development, while steroid components in root exudates act as signaling molecules, influencing rhizosphere microbial composition and regulating plant–microbe interactions.

Furthermore, heat map analysis showed that the secretion of lipids (e.g., Austalide L, Myxopyronin A) reduced the enrichment of bacterial colonies such as *Subgroup_7* and *Haliangium*, while Litseakolide A and 9,12,13-Todea enhanced their enrichment. Organic acids (e.g., Azelaic acid, 3-(3-hydroxyphenyl)propanoic acid) exhibited the opposite effect. KEGG enrichment analysis revealed significant enrichment of nucleotide metabolism, purine metabolism, and the ABC transporter pathway (within Environmental Information Processing) across treatment groups. Rhizosphere microorganisms support proliferation through nucleotide synthesis (e.g., adenosine and guanosine nucleotides)—for instance, D-ribose transport, mediated by the RbsA enzyme, represents a critical step in microbial reproduction (Abulfaraj et al., 2024), while secreted ribose functions as a plant symbiosis signal (Keren et al., 2024). Purine metabolism generates cAMP via the cascade AMP → ADP → ATP, directly influencing microbial energy metabolism efficiency (Kai Wang et al., 2016). ABC transporters participate in nutrient transmembrane transport (e.g., amino acids) and microbial toxin efflux—e.g., Aeromonas hydrophila alters outer membrane phospholipid transport through MlaF gene mutations to enhance antibiotic resistance (Powers and Trent, 2018). Furthermore, ABC transporters are implicated in plant hormone signaling (e.g., IAA). This association potentially underpins a mutualistic relationship characterized by bidirectional resource exchange: microbes secrete auxins, while plants provide carbon sources. This establishes a cyclical “microbe-secreted auxin–plant-provided carbon source” relationship (Wang, 2017).

The T6 treatment (mixed strains) enriched more pathways—including nucleotide metabolism and glycerophospholipid metabolism—than single strains. Nucleotide synthesis supports rapid microbial proliferation, while adjustments in membrane lipid composition (e.g., upregulated phosphatidylethanolamine) enhance environmental adaptability (Zhao et al., 2021; Abulfaraj et al., 2024), providing metabolic evidence for why mixed bacterial agents outperform single strains.

## 5 Conclusions

**Figure 6:**
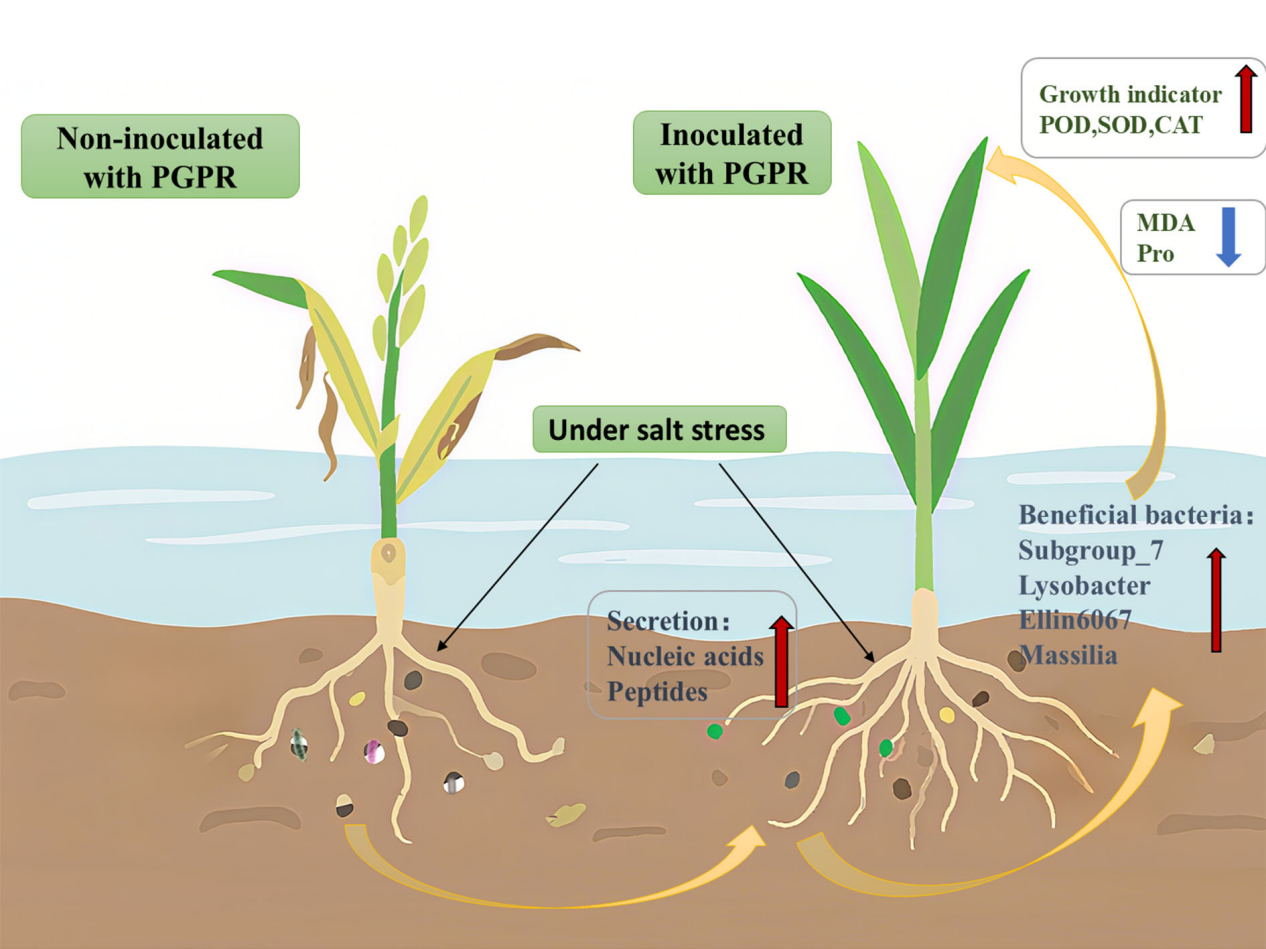
Effect of Plant Growth-promoting Rhizobacteria on the Growth of Rice Seedlings under Salt Stress and Its Microbiological Mechanism. This illustration demonstrates the effects of PGPR inoculation on rice cultivated in saline-stressed soil. The left panel depicts untreated rice plants and their root systems, where the native microbial community remains unaffected by exogenous microorganisms under chronic salt stress, resulting in significant growth inhibition. In contrast, the right panel shows PGPR-inoculated rice and associated roots. Inoculation stimulated the secretion of specific rhizosphere metabolites, which effectively recruited beneficial microbiota and restructured the microbial community composition. Consequently, soil microbial diversity was enhanced, concomitant with marked improvements in rice growth indices. These synergistic effects collectively promoted plant development and stress adaptation.

This study demonstrates that PGPR inoculation significantly enhances root exudate production in rice, and these exudates subsequently recruit beneficial microbiota (*Subgroup_7*, *Lysobacter*, *Massilia*) that promote plant growth and development (**Figure 6**).

These findings establish a PGPR-rice metabolic mutualism model wherein PGPR recruit stress-protective microbiomes while synchronously tailoring root exudates to optimize nutrient acquisition and ROS scavenging. The enhanced performance of the T6 consortium highlights the potential of synthetic microbial communities for reclaiming saline soils. Future research should validate these mechanisms in field trials and integrate transcriptomics to elucidate underlying gene regulatory networks.

## Data Availability

The original contributions presented in the study are included in the article/**Supplementary Material**. Further inquiries can be directed to the corresponding authors.
